# Beyond Participation: Politics, Incommensurability and the Emergence of Mental Health Service Users’ Activism in Chile

**DOI:** 10.1007/s11013-018-9576-9

**Published:** 2018-04-24

**Authors:** Cristian R. Montenegro

**Affiliations:** Department of Methodology, London School of Economics and Political Sciences, Houghton Street, London, WC2A 2AE UK

**Keywords:** Service user organisations, Mental health systems, Politics, Reflexivity, Incommensurability

## Abstract

Although the organisation of mental health service users and ex-users in Latin America is a recent and under-researched phenomenon, global calls for their involvement in policy have penetrated national agendas, shaping definitions and expectations about their role in mental health systems. In this context, how such groups react to these expectations and define their own goals, strategies and partnerships can reveal the specificity of the “user movement” in Chile and Latin America. This study draws on Jacques Rancière’s theorisation of “police order” and “politics” to understand the emergence of users’ collective identity and activism, highlighting the role of practices of disengagement and rejection. It is based on interviews and participant observation with a collective of users, ex-users and professionals in Chile. The findings show how the group’s aims and self-understandings evolved through hesitations and reflexive engagements with the legal system, the mental health system, and wider society. The notion of a “politics of incommensurability” is proposed to thread together a reflexive rejection of external expectations and definitions and the development of a sense of being “outside” of the intelligibility of the mental health system and its frameworks of observation and proximity. This incommensurability problematises a technical definition of users’ presence and influence and the generalisation of abstract parameters of engagement, calling for approaches that address how these groups constitute themselves meaningfully in specific situations.

## Introduction

### A Voice Out of Place

It was 3 days after I returned to Chile for fieldwork. A colleague invited me to a seminar called “Stigma towards Mental Illness: A Public Health Challenge”, hosted by the historic Dr. Salvador Allende School of Public Health. It was the second event of its kind, and the focus was placed on concrete policy recommendations through case studies coming from different experiences in Chile and abroad.

The programme included keynote presentations by international and local experts, and results from a pilot study on peer-support strategies, developed in Santiago. Inside the large auditorium, there were around 400 people, mostly professionals working in the public system looking for innovations and ideas, but there were also academics, students and representatives from NGOs and community organisations. The first presentation was given by a North American expert, speaking in Spanish with slides in English. When it finished the audience was invited to ask questions.

Ramon, an activist and ex-user that I was supposed to meet that day, raised his hand first. He introduced himself as “a mad person”, causing surprise and smiles. He criticised the use of English in a seminar given to a Spanish-speaking audience, and then he questioned the intentions of the presentation, asking to what extent anti-stigma campaigns, and the idea that mental health conditions are just like any other condition, relied on a reductionist biomedical conception of suffering and was a way for the pharmaceutical industry to make their products more acceptable for the population.

The organisers, seated close to me, were clearly uncomfortable with the situation. Their discomfort was compounded by the resounding applause that Ramon received after finishing his observations, with people saying “that is true”, “he’s right” or “he’s very brave!”. The presenter apologised for using English and attempted to demonstrate his user-movement “credentials”, highlighting his long-term work with user organisations in New York. The following presenters picked up the theme, expressing in different ways that purely medical perspectives had limits and that more voices needed to be included, especially the voice of users. It was striking to see how, suddenly, the voice of “the mad” mattered, how a presence became perceptible, forcing the experts to make some room.

The last presentation of the day described a local pilot study testing the Critical-Time Intervention (CTI) model, a peer-support strategy developed in New York during the mid-1980’s to reduce rehospitalisation rates after discharge (Susser et al. 1997). The presentation included testimonies from users working as peers. As soon as it finished, Ramon raised his hand again. Instead of praising the involvement of users he asked about the earning gap between them and professionals in the pilot, stating that participation makes no sense if it is on the basis of unfair and paternalistic working conditions.

Nobody seemed eager to address the question and there was no applause. People near me said “does that really matter?”, “isn’t that too much?” or “he’s just a *chaquetero*^1^”. A user working in the pilot replied to Ramon saying that the money was fine for him and that being a peer in the project was far more important than the income. He received the applause this time, other questions came and the seminar moved on. But what does such change reveal?

Although controversial, the first remarks expressed a sense of rebellion that appealed to the audience and shifted the subsequent tone of the meeting, itself a microcosm of the mental health field and its main publics. Ramon’s voice was given a place. But when he interrogated the very role of users and the terms and conditions defining their “participation”, he both transgressed and revealed the limits of that place. His controversial perspective was accepted and supported, but he was not expected to question the role that was already afforded to him. Paradoxically, the mad person lost his role when he reflexively questioned it.

For Jacques Rancière what makes a conflict *political* is not a clash of interests or perspectives but a disagreement about the legitimate parties in the conflict itself. The tension contained in Ramon’s second question could not be reincorporated into the meeting as a “perspective” because it expressed a clash between logics, between ways of defining who had a part and on what terms. The question introduced an incommensurability to the apparently unified horizon of the meeting, an outside that revealed the meeting’s “public transcript”, that is, the frame of expectations and the order of roles constituting it as a social space (Scott 1990). Only from the outside could the meeting itself be observed as a contingent scheme of roles and asymmetries. Only through excommunication could the mad person communicate himself.

This article examines the way activists and user groups develop a vision of themselves and dispute their definition in front of other agents and their logics. In the following section, the global and local framings around users’ collective actions are described and the literature from the social sciences is considered, in order to re-specify the aim of the article.

### Emergence Between Global Calls and Local Expectations

In South America, the organisation of mental health user groups and their involvement in policy and/or activism constitutes a recent and under-researched phenomenon. While the call for users’ involvement is common in mental health plans and in declarations from authorities, user groups are generally placed within an undifferentiated “civil society” (Montenegro and Cornish 2017), whose role is to support specific reforms in the region (Ceriani, Obiols, and Stolkiner 2010; Montalbán 2013; Zaldúa et al. 2012).

On a global scale, several agents have called for the empowerment and involvement of user organisations at all levels of policy making. The World Health Organisation’s new Mental Health Action Plan 2013–2020 has identified new constituencies and leadership roles in the field, within and across countries, including users’ organisations, described as crucial agents with a stake in policy, and calling for national mental health systems to strengthen them (2013:12). At the same time, the Convention on the Rights of Persons with Disabilities (CRPD) has given unprecedented centrality to disabled people’s organisations, including persons with psychosocial disabilities (Minkowitz 2013). Traditional organisations in the field have also developed guidelines on working with user organisations (Wallcraft et al. 2011), identifying them as a key ally in tackling stigma and discrimination.

But studies coming from the social sciences—including user/survivor research-problematise the technocratic framework of these global calls, tracing the political and institutional forces shaping the organising efforts of users and survivors and highlighting the power imbalances that limit their scope of action (Barnes 1999a, b; Beresford 2010; Brosnan 2012; Carr 2007; Lewis 2014). Users’ advocacy efforts are linked with broader social, cultural and political dynamics that transcend the mental health system’s declared interests or expectations (Crossley 2006a; Everett 2000; Morrison 2005), interests that, in most cases, constitute the very objects of contention and dispute in the hands of user groups. That means that the actual organisation of users cannot be simply seen as an implementable “feature” within a technical definition of mental health systems, or deduced from a normative vision of who is or who should be an agent in the field.

Generally, these analyses trace the organising efforts of user groups as they emerged and consolidated in the English-speaking world, where such practices have a relatively long history (Campbell 1996; Crossley 2005, 2006b). In Chile, as in most of South America, there is no “original” user movement serving as a standard to understand variations over time, or to estimate and theorise the effects of broader social, political or economic processes. As revealed by Ramon’s intervention, the role of users and the meaning of participation are at stake, with no clear definition coming from policy (Contandriopoulos 2004).

In order to understand the specific politics of service-user activism in a new context, and the way a collective identity is produced and projected into society, a more abstract conceptualisation of power and resistance becomes necessary, one that can guide the ethnographic unpacking of positions and identities. Rancière’s (1999) distinction between “politics” and “police” represents a valuable alternative.

Although developed in the context of a complex discussion against political philosophy, Rancière’s work has gained traction in the analysis of the politics of marginalised groups (Dornhof 2011; Jazeel 2015; Klee 2013; May 2008). For Rancière, “police” is, fundamentally, the practice of matching groups to functions and activities, or the identification of groups according to the function they accomplish in any given “community”. “Police” proceeds as a determination of what each group “is”. (…) “it is an order of the visible and sayable that sees that a particular activity is visible or not, that this speech is understood as discourse and another as noise” (Rancière 1999:29).

“The essence of the police is to be a partition of the sensible characterised by the absence of a void or a supplement: society consists of groups dedicated to specific modes of action, in places where these occupations are exercised, in modes of being corresponding to these occupations and these places. In this fittingness of functions, places, and ways of being, there is no place for a void. It is this exclusion of what ‘there is not’ that is the police-principle” (Rancière 2010:21).

In the face of police runs a counterforce, ‘politics’, a struggle against the distribution of parts and roles, announcing the gap between beings, places, and functions from a position that is not yet distributed, from what does not fit, from a void. Politics is “the production of a series of actions of a body and a capacity for enunciation not previously identifiable within a given field of experience, whose identification is thus part of the reconfiguration of the field of experience” (Rancière 1999:35). In Connor’s interpretation, “politics is an interruption into the realm of what exists, in its divisions and parts” (2014:11).

During the seminar on stigma, Ramon’s final words came from a place that did not have a place, revealing a capacity that was not yet visible, a capacity to reflect upon its own role and value. As a carrier of a vision of himself, he became “excessive”, beyond the boundaries of the “acceptable” mad voice. But how do activists collectively reject expectations of policy? How do they create a place of their own?

This study looks at the politics of the mad in the context of the ordering forces of “police”: the actions through which organised users in Chile reflexively shape their own collective identity and dispute their own position. Based on ethnographic fieldwork with Ramon’s organisation, it considers the practices through which user groups orient themselves, project themselves and sustain their own difference against other agents’ and systems’ frameworks of legibility and approachability.

## Methods

There are multiple expectations about the role of user groups in the mental health field, influenced by global calls and international examples, mobilising agendas and opportunities for users but also shaping a definition of their role and contribution. Methodologically the challenge is to get closer to the organised efforts of users and see how they read such complex environment. A focused ethnography (Knoblauch 2005) was chosen as a research strategy, focused precisely on how one such group projected itself in the field and defined its own meaning and goals. The ability to focus the ethnographic attention upon specific aspects and events relies on the researcher’s accumulated experience and expertise in their field (Knoblauch 2005).

Before starting the fieldwork, I contacted currently active user-led initiatives without the mediation of local and national health authorities or professionals. This was partly facilitated by prior links with members of those organisations through the years in my position as a social researcher and academic within the Chilean mental health field. Three organisations were initially contacted: the National Association of Users of Mental Health Services (ANUSSAM), “Radio Diferencia” (RD) and “Agrupación Libre-Mente” (ALM).

ANUSSAM, the oldest user organisation in the country, was born in 2000 out of a confluence of interests between the Mental Health Department of the national Ministry of Health and a group of users participating in CORFAUSAM, a coalition of family organisations (Montenegro and Cornish 2017). In the typology of the mental health system, ANUSSAM is the only organisation representing the interest of users at a national level. ANUSSAM was ruled out for this study because the frequency of meetings and activities during that time was very sparse and strictly linked to the formal need to select a new board. There were no other plans or relevant activities and even during those few meetings only a fraction of the members participated.

Radio Diferencia is a radiophonic project born in 2005 led by a group of users of the El Salvador psychiatric hospital in Valparaiso. Defining themselves as “the voice of those without a voice”, it produces several radio shows with different thematic segments led by users, with the aim of educating the public about the rights of persons with psychiatric conditions, in order to dispel the myths around mental illness. They usually invite authorities, activists and all sorts of experts to speak on the show: The first time I met them I immediately and unexpectedly became an interviewee. However the activities of RD were strictly related to the production of the show. Moreover, the fact that the show was produced in the facilities of a psychiatric hospital further limited their range of opinions and oppositions. The most relevant insights came from individual conversations with their members after the show was over.

ALM was the only group who explicitly organised their meetings to be open to anyone. They were engaged in a series of relevant activities that multiplied the possibilities of engagement beyond the meetings, such as demonstrations, participation in events, meetings with other groups, etc. The more I was exposed to their activities and plans, the better I came to capture a sense of continuity and maturation. Gradually, during the course of fieldwork I decided to follow them closer, wrapping the research project around them.

Concretely I participated in 17 meetings with ALM and I joined them in several informal activities. That includes regular weekly meetings (9), extraordinary meetings -focused on specific projects- (2) and events and activities where members acted as representatives of the group, together with other user groups and supporters (6). It amounted to approximately 70 h. In addition, I conducted personal interviews with five users engaged in the group (5). Field notes recorded the conversations that took place during the meetings, focusing on how the group described its own goals and positioned itself in front of other, relevant agents and agendas. In parallel, I closely followed the posts and debates produced by the group through their Facebook page, before and after fieldwork, maintaining regular online contact with some of its members to this day. I also participated in meetings and activities with the other two organisations (7) conducting interviews with their members (10), which helped me to better situate the position of ALM in a larger and diverse network of activists.

Fieldnotes, audio recordings and transcriptions from interviews were integrated into a qualitative software package (MAXQDA12) to assist the analysis. Thematic analysis was used upon these sources, with a coding framework combining deductive and inductive themes. Participant observation had precedence in the analysis and in the findings, and therefore the results follow a sequence of activities that concentrated the energies and reflections of the group during fieldwork. These activities linked the group to (i) the legal system, (ii) the mental health system and (iii) society at large. After first introducing the characteristics of the group, the findings section deals with each of these realms.

Written consent was received before every individual interview. In the case of the meetings, their composition changed permanently, therefore at the start of each meeting I made my aims explicit to old and new members. The group fully supported my project during the process, and I was granted permission to audio-record most of the meetings, enhancing the analysis of field notes, in line with the principles of focused ethnography (Knoblauch 2005; Wall 2014).

The focus of the project is on how the group creatively and reflexively negotiated its collective identity, regardless of each member’s specific background and/or circumstances. The main insights come through open, spontaneous discussions that involved most of the members, both old and new. Although aware of my aims, due to the changing composition of the meetings, not all the participants gave written consent to be identified in my research. Therefore, both for analytical and ethical reasons pseudonyms are used in the article.

## Findings

### The Group

In early 2015, I got in touch via email with Agrupación Libre Mente through Ramon, an ex-user and disability rights activist whom I had met in 2014, in the context of my involvement in the Quality Rights project where professionals and users evaluated mental health facilities across the country (Minoletti et al. 2015). He discussed my project with the other members and he replied that they were OK with me coming to their meetings. I arrived in Santiago by the end of July 2015 and 3 days later I attended the previously described seminar. I did not know Ramon was going to be there.

That day, during lunch, I approached him and Claudio, another member of ALM, and we talked for a while before other people circled them to ask them questions. It was Monday, the day ALM met, so they invited me. The meetings were held on the second floor of Librería Proyección, a busy anarchist bookstore located on the side of the colonial San Francisco church, in the heart of the capital.

Libre Mente was born in 2013. The group resulted from the transformation of a prior “auto-formación” [self-training] initiative led by psychologists and other young professionals. It was directly connected with the work of the Centro de Acción Crítica en Salud Mental [Center for Critical Action in Mental Health], an active group within the burgeoning “anti-psy” scene in the country. Antipsychiatry and “anti-psychology” have had a resurgence, particularly in academic psychology, through the work of Chilean philosopher Perez-Soto (2014) among others. This Marxist, anti-capitalist form of anti-psychiatry resonated with the values of a generation of students that participated in the waves of protest sweeping the country over the last decades (Cabalin 2012).

Ramon, an active member of the disability-rights scene in the country, was the first member of the group with “a direct experience of psychiatrisation” (his words). Although his aim was to shift the group towards a user and ex/user based initiative, for him, service-user exclusivity was pointless: the group saw itself as the outcome of solidarities across the user/non-user divide, as in other Latin American countries where mental health activism is diverse by definition (Freitas 2011). This is why initially the group called itself a “Movimiento de Personas por la Salud Mental” [A People’s Movement For Mental Health].

Through 2014 Ramon also formed the “Locos por Nuestros Derechos” [Mad for our Rights] collective, an advocacy initiative responsible for the Manual de Derechos Humanos en Salud Mental [Human Rights in Mental Health Handbook] (Locos por Nuestros Derechos 2015). He had already visited mental health services, universities and diverse community organisations across the country and internationally, disseminating El Manual and offering his critical views around forced and irreversible treatments, the medicalisation of children’s behaviour, the role of pharmaceuticals in influencing policy and the unacceptable complicity of professionals. Users, students and professionals generally wanted to know more and in response an open invitation was extended to ALM’s meetings.

The diffusion of El Manual prompted a circulation of participants into the meetings, mostly psychology, social work and occupational therapy students, journalists, social scientists, community organisers and activists interested in the group. During fieldwork the number of participants in a meeting fluctuated from 7 to 15. Amongst the permanent participants, there was a group who did not describe themselves as users, ex-users or survivors. Most of them worked in mental health or related fields, in different levels and locations, and some of them had a longstanding connection with LGBT activism, the student movement or animal rights advocacy. It was a diverse group in terms of age, gender and background, but they all had a wide knowledge of mental health policies, the power of the pharmaceutical industry and the damage that tradition mental health services could do.

There was also a permanent group of users with whom I engaged the most. I interviewed each of them during the 5 weeks of fieldwork. Renata and Pedro were living together for about a month. Ramon lived with his partner and worked independently in construction, while Pedro and Claudio had met in psychiatric facilities and together sold different products in central Santiago’s street markets. Alonso lived with his family and had a job through the intermediation of a local disability office. All of them were acquainted with other users through their paths across institutions and rehabilitation services, inviting them to the meetings.

Almost all of the users had experienced neglect, abuse and manipulation in the hands of psychiatrists and other professionals working in the mental health system. Some had quit all medication, particularly Ramon and Claudio. Alonso was working towards discontinuation, with the advice and support of the group. Yet others, like Renata, openly defended the informed use of psychopharmacological solutions. Among them Ramon had the most distinctively radical stance towards traditional mental health services, using his own life experience to publicly speak against psychiatric abuses. Not all of them shared his vision or intensity. While their stories overlapped, the group had no unified aims.

Each meeting was started by Ramon or other members briefly describing the last meeting’s agreements or issues that required follow-up. Then introductions came by new and old members, accompanied by lengthy conversations. The level of attention given to each participant, regardless of how long they had been participating always surprised me, since the moment I introduced myself. More importantly, the diversity of the meetings, with users, ex-users, non-users and uninitiated guests produced a highly deliberative space, where the definition of the group’s aims and identity emerged reflexively (Archer 2007), as an ongoing achievement rather than a starting point. Every meeting re-started the group.

But regardless of the changing composition of each meeting and the countless interesting topics and situations observed, there were three specific projects that demanded more time and energy and required the group to decide on a number of important issues. These projects forced the group to reflect upon its own identity, that is, on how it was perceived by a set of others, others made relevant by the projects. These three projects provide the ‘focus’ for the focused ethnography. These were the project for the creation of a coffee shop, the parallel creation of a new national mental health plan, and the planning of the first Mad Pride Parade in Chile. These projects, in turn, involved a process of engagement with the legal system, the mental health system and society as a whole.

### The Legal System

There were 8 participants at my first meeting including other first-timers. Usually, each new participant was subjected to questions coming from all members. The group interrogated me, demanding more than a repetition of what Ramon had already told them, and I could immediately perceive the importance of testimony and position. The other first-timer was Alonso, a service-user, and he was interrogated about his diagnosis, pharmacological treatment, services being received, interaction with professionals, etc. These extended personal introductions created a sense of presence: we were not “just” there, to observe or learn, we were part of the meeting with stories and concerns potentially linking us together.

I offered my help with activities. At that point, their main project was the creation of their own coffee shop. Over the next 4 meetings this was the main topic of discussion and planning.

During the first two encounters, the conversation around the project was creative and playful. In the “imagined” coffee shop every idea made sense, from the most trivial business considerations to the ambitious desire to “rehumanise the normals” through the cafe [Renata, Woman, 45 y/o, User.]. In contrast to what they viewed as an alienated, individualistic and sad society, the group wanted to create a “café con-ciencia” [wordplay meaning “consciousness coffee” and “coffee with science”], where clients could change not only their opinions about madness but could also experience a sense of personal transformation, the sense of being in the difference.

As such, beside the relevant economic benefits, the project expressed a desire for a meaningful re-engagement with society, reversing stories of miscommunication and alienation: “This should be a space of expression, not only our expression but the expressions of those who come here (…) It gives us the possibility of listening to the other, in this case, the client, and this is similar to what happened with us and psychiatry because… you go to the doctor and you want to be heard and he says ‘ok, time’s up’ and that’s it” [Claudio, man, 55 y/o, ex-user].

Through their plans for the project the group rehearsed notions of shared decision making, horizontality, transparency and democracy. The shop was an imaginary space for them to play with their possibilities of existence as a group. But transforming the utopia into a real place required the adoption of a form. For the project to work, it had to be legally valid, and for this, the group itself needed a personalidad jurídica, a legal persona, acceptable by the legal system as a right-and-duty-bearing unit (Dewey 1926).

It was a big step, so the group sought some technical advice. A coffee-shop owner, an expert in cooperatives and a lawyer came to the meetings. Some members had already created a “corporation” that could be reactivated, a type of legal persona that allowed them to apply for funding, conduct research, run businesses, etc. Although comprehensive, the corporation required an inflexible set of internal functions and distinctions: between board and associates; president, secretary and treasurer; normal and extraordinary sessions, etc. Another legal persona, the “cooperative” seemed to better match the self-image of the group, with shared decision-making and equal distribution of work and income. But such form could only be used for very narrow purposes, preventing the pursuit of broader “social” goals and eliminating the possibility of receiving funding from external agents, such as the State, international agencies and NGOs.

“Once you make a decision about your aims, it will be easier for me to give advice on the best juridical personality” was one of the final remarks made by Hector, an impeccably dressed lawyer invited to the last coffee-shop related meeting. But the selection of a legal form was not a straightforward decision. The proposed project symbolised the group itself, it was an image of how they wanted to be seen, and a way to “come back” to society on their own terms. But to be viable it required a legal fiction, legible by the legal system, alien to the ongoing self-identification of the group. The group faced a paradox, a potentially endless oscillation between options (Perez and Teubner 2006): the only way to be what they wanted to be -a financially viable, user-run coffee shop- was to be something they rejected -a corporation with hierarchical roles and internal divisions.

For Teubner, “Real paradoxes are highly ambivalent. They contain destructive, paralysing potentials but contain at the same time productive, creative possibilities” (2006:48). During the last part of the meeting Claudio made this point:

So, how to say it, these meetings like the one we’re having right now, while not part of a formal legal figure, these meetings place us in a relation, they make us develop a relationship between each other, on another level.

The closer the group got to this dilemma of identification, the stronger the need to acknowledge its own relational reality as already there, regardless of legal identifications. As expressed by Cooper in her ethnography of mental health courts in the San Francisco Bay Area, USA, “The court’s formulation of jurisdiction and its creation of individuated subjects reach an impasse at the moment of the social” (2018:100). In the context of activism, the pre-existence of social bonds provides a foundation for the group to navigate the options of legal identification.

We will return to the fate of this project in the final section of these findings.

### The Mental Health System

During my fifth meeting with the group, several professionals mentioned the ongoing elaboration of a new national mental health plan. The plan considered a process of country-wide consultation among every level of attention in the public mental health system, including civil society organisations. As an growingly active and visible group in the field, Libre Mente was expected to participate.

As I learned from the person in charge of this plan at a national level, the design of the consultation was very simple. The Mental Health Department of the Ministry of Health sent a draft version and each involved group or agent could discuss it collectively, adding new sections and feeding this back to the Department. Participation in the elaboration of the plan meant engaging in this feedback process, but there were no guarantees or rationale for how this information would be incorporated into the final plan.

Conversations about this consultation re-emerged in several meetings and in the context of different discussions in the group. The issue was originally raised by two professionals who had already participated in the consultation, and by Elisa, a user who was very active in her own Consejo de Desarrollo Local (2) where she learned about the plan. Ramon, on the other hand, had a different view:

“Personally I don’t want to participate in whatever the mental health department wants to do. I mean, because that plan is already decided. What underlies this exercise is the same old approach, four or five psychiatrists, who already monopolise all the decisions, make a deal. The only thing they want is money for more drugs and treatments, they disguise that with the discourse of rights and users’ participation… In the group making the real decisions there are no users, but they want to validate their plan so some users will share their views, and that’s why for example Eva who leads an organisation in Puente Alto, is inviting Elisa who lives on the other side of Santiago so that she can show up as one more user participating and validating the whole thing”.

Elisa was a middle-aged woman struggling with her own diagnosis and the sole provider of care for her disabled husband. She was an active member of a “Local Development Council^2^”, the main mechanism of citizen participation available within her health service, and she had successfully brought herself to the attention of care providers, receiving urgently required treatment. El que no llora, no mama [“The crying baby gets the milk”] was her (and many others’) leitmotif, and the plan’s participatory process was an opportunity to cry and be heard. She confronted Ramon saying that she was the one interested in the process, and she had contacted other persons to look for possibilities of involvement.

But for Ramon there was a deeper concern: “Who created the first draft? Why them? Why are they supposed to know better? (…) they choose what we’re supposed to discuss, ‘discuss this and that’, what are the concerns, the problems, what are the gaps, etc. They choose the topics, the problems and the words. They choose who’s invited to give comments on the draft. They choose what to include from the feedback, they write the final plan and on top of that, they take the credit for being inclusive and participatory!”. In the words of Claudio: “The problem is that we are just reacting to what they are doing, we should work on the basis of our own work (…) because when we start to engage in a fight with the institution we forget about ourselves”. Rejecting the plan was a way for the group to confirm its own autonomy and value.

Increasingly, the conversation moved into the relation between ALM and the mental health system. For some, the flawed methodology and the dubious intentions of the Plan were precisely the reason why the group had to engage. “We should at least define what do we expect from the mental health system, what’s our ideal”, said one of the professionals. For him and others, the group needed ambitions beyond itself. In the metaphorical expression of Valeria [Woman, Psychologist, 30 y/o], while it was fine to raise the pins every time the ball knocked them down, eventually the whole game had to be transformed. The ultimate goal was to transform the mental health system, and not just to help each other to deal with their problems. Ramon responded to this and similar concerns:

Instead of thinking about ourselves working with the institutions, our plan is to empower ourselves, to define ourselves, to be an agent of change in ourselves, more than waiting in the system because the system is oriented to reproduce itself, and it only works for its own interests (…) So the change has to do with ourselves, how we build a citizen in ourselves, empowered, and we empower other people (…) That’s our mental health plan, that’s what we need to build. They care about facilities, budgets, drugs, professionals, that’s all. They call it ‘human rights’, they say ‘inclusion’ because they have to do it, but those are just names.

So instead of changing the game they could rather create and play their own game. For Claudio, “It’s good when we share stories about us helping others, our own reactions to injustice. To the extent to which we as a collective create forms of action based on dialogue and discussion, not like those arbitrary and abstract plans that come from the outside. The advantage in that you start from your reality, not from suppositions about what we, I mean, they need.”

The invitation from the mental health system created an opportunity to paradoxically take distance from that system. By seeing the mental health system as a blind, self-reproducing machine and its participatory plan as arbitrary and “exterior”, the group reflexively recognised its own exteriority in relation to the system. The “either/or” hesitation between participating or not led to the realisation of their own existence beyond (or outside of) the options, beyond participation.

The plan and the process of consultation required a sequence of activities and a certain temporality (Renedo and Marston 2015), guiding the actions of the mental health system during a predefined set of years, in line with WHO’s and PAHO’s health plans for the region (Caldas de Almeida 2005; Minoletti, Sepúlveda, and Horvitz-Lennon 2012). If policy plans encode visions of the possible and the desirable (Abram and Weszkalnys 2011), then what was rejected was also the temporal determination of the possible and desirable for the group. The plan became observable as a technocratic chronology in relation to which they could embrace “their own plan” for self-transformation, their own notions of betterment and progress, their own distinction between past and future.

For Abram and Weszkalnys (2011) the power of plans relates to their ability to draw different publics into a sequence of actions. In our case, the National Mental Health Plan’s aim was also to guide the action of a whole series of agents constituting the system’s relevant environment. Rejecting the plan was also a way to avoid a place within that environment. This third process is clearly visible in the next section on the mad pride parade.

### Society

Gradually, a decision about the ‘legal personality’ for the coffee shop became difficult to make. As stated before, the problem was not so much about which legal form to take, but about the need to take one in order to be what the group wanted to be. On the other hand, with its dependency on opaque legal definitions and administrative procedures outside of the group’s scope of action, the project was naturally replaced by initiatives that actualised a sense of control, completion and progress. The main new activity was the organisation of the first Mad Pride Parade in the country, La Marcha del Orgullo Loco.^3^

Some participants knew about international versions of the parade. Locally, the Parade for Sexual Diversity originally called the “Gay and Lesbian Pride Parade” mobilised tens of thousands of people every year since its origin in 1999 (MUMS Chile, n.d.). At the same time, recent waves of student mobilisation in the country widely deployed theatrical and carnivalesque resources as a tool to communicate demands of social transformation (del Campo 2016), providing a relevant symbolic background.

The parade’s planning and preparation required many activities and decisions. While Ramon led the process, different tasks were distributed across participants, and all decisions were discussed in the Monday meetings and other extraordinary sessions. The first decision concerned the dates and place. The parade involved blocking key avenues in central Santiago, and a request had to be submitted to the Municipal Authority.

The initial plan was to organise the parade as a counter-manifestation to the International Mental Health Day (10th of October), celebrated since 1992 and initiated by the World Federation for Mental Health to rise the publics’ attention towards mental health issues (Brody 2004). But while a counter-manifestation could enhance the visibility of the group and its claims, For Drago, a user and university student “(…) this manifestation should come from us, not from what they are doing (…) otherwise it would look like we are simply reacting to what they’re doing”. Finally the date was moved to November.

A fixed date and venue were required to start inviting as many people as possible. But who was going to be invited? The mental health system already had a recurrent “public” (Newman 2009) assembled around the perennial call for financial resources (Montenegro and Cornish 2017), a call linking global and national agents from the PAHO, INGOs, local NGOs, Family Organisations, and many others. The group rejected this call, considering itself outside of the instrumental version of the public sphere created by the mental health system. Who was represented in the parade then? Only ALM?

Concretely, the initial concern was about who had to be rejected. Political campaigners could use the parade for self-promotion. They had to be excluded or required to refrain from using banners or other messages. But other groups were harder to distinguish, especially NGOs and established family organisations. Ramon suggested that all participants should sign up to a document, adhering to certain principles. The group discarded this impractical. Others suggested installing a “press point” where journalists, where people from the media and/or any curious people could ask questions and receive agreed-upon information, reducing the risk of both misrepresentation and misinterpretation. Connected with this idea, Julia [Woman, caregiver, activist, 38] suggested a manifesto with the group principles, “a text that defines who we are and what we want, and well if you disagree you better stay at home”.

Questions about how to present themselves came back to the conversation. But while past hesitations reflexively produced a sense of distance, the nature of the parade required intense engagement and exposure. Not all the interests could be controlled in advance, especially as they unfolded in space, with the multiplicity and simultaneity of voices and orientations that this implies (Massey 1999). Furthermore, the parade had the power to irreversibly situate ALM in front of other users, professionals, NGOs, the media and society at large. The stakes were high and I could feel how a relentless preoccupation with integrity and autonomy met an equally relentless drive towards the outside, towards the streets, towards the other. How could the group navigate this tension?

Right from the beginning, planning the parade was followed by a secondary, less practical reflection about madness itself. Faced with the question of self-presentation, that reflection became central. Are we mad? Are we proud of it? Is this the word we want to use? Is it actually offensive? For a user in another organisation, celebrating “madness” was like giving up the battle against prejudice, like saying “ok, you won, you can call us whatever you want” [Esteban., 27, user]. Even in Libre Mente the issue was not settled. For some the point was to re-signify madness, focusing on the other meanings of loco: Radically original, extraordinary, unpredictable, out of this world, etc.

But Julia’s position pointed to another function for the word: “The thing about ‘mad’ has to do with who calls you like that. It is one thing when others call you mad, and a different thing if you do it yourself and you do it with pride”. As such, beyond semantics, the word delineates a community for the first time, the community of those who are not ashamed of calling themselves mad. This was not the “population suffering from a mental health condition” or the “group of people living with a psychiatric disability”. Not even the “representatives from service-user organisations” summoned by a contingent policy requirement.

“Mad” is the word that the mental health field rejects as an expression of ignorance and prejudice. To a certain extent the field itself is founded on this rejection, including groups generally conflated with users, such as family members and caregivers. “Your son is not mad, he has a mental illness and there is a treatment” is the statement that constitutes them as caregivers, dispelling any doubt and setting a course for their lives (Montenegro and Cornish 2015). They, as Claudio and Renata expressed, felt clearly insulted by the name of the parade. But, precisely because of this, embracing “madness” had the potential to create the kind of alliances and solidarities they wanted to create, to project and protect their difference in the ecology of interests populating the parade, to create a separation, a sphere of validity and expression, incommensurable to the field.

## Conclusions

Libre Mente’s process of self-differentiation is expressed through instances of hesitation linked with practical activities and aims. These hesitations reveal a practice of collective reflexivity that threads together an orientation towards social transformation and a recognition of the group’s value, allowing them to affirm themselves against parameters of proximity and engagement coming from the outside, and to see themselves as incommensurable to those frameworks. Rejecting the legal persona, refusing to engage in the policy consultations and embracing the apparently offensive notion of ‘loco’ are not just actions chosen out of a coherent pool of options. These decisions shape the group itself, its visibility and compatibility with other expectations and agendas. Broader consequences are elaborated in the following section.

### Activism as a Practice of Reflexivity

With few exceptions (e.g. Noorani 2013) the literature on activism in the mental health field has not placed a strong emphasis on reflexivity. But, as seen in the findings, conversations about themselves and their role constituted an important tool for the group. As stated by Archer in her analysis of collective agency,

“One of the main tasks that reflexive deliberations perform is to enable subjects to consider their concerns in relation to their social circumstances and their circumstances in the light of their concerns, to revise both accordingly and then to think of their future courses of action in terms of the revisions made” (2013:151).

What the findings reveal is precisely how a series of circumstances required the group to consider its own value and meaning, as a ground from which to observe those circumstances. Reflexivity was a practical tool, a mechanism to overcome dilemmas and paradoxes steaming from external requests. This self-recognition informed different decisions and shaped an ability to make distinctions and to select alliances, invitations and forms of self-presentation. Using systemic terminology, a self-referential tendency (Luhmann 1995) is the condition of possibility of forms of communication and engagement, a mechanism for the group to not be dissolved in the contingency of interests and frames, expectations and roles defining the field.

#### A Politics of Incommensurability

Literature on organised activism in mental health has tended to focus on processes of engagement with and influence upon policy processes: Its preeminent concern is the interaction between affected groups and institutions or powerful agents. Critical studies around activism in mental health give attention to logics of professionalisation (El Enany, Currie, and Lockett 2013; Harrison and Mort 1998) and co-option (Pilgrim 2005), but still views them as distortions and deviations from an otherwise desirable growth in engagement, influence and “proximity” (Bacqué, Rey, and Sintomer 2004).

This paper, in contrast, demonstrates how practices of disengagement and distancing, what Papadopoulos, Stephenson and Tsianos (2008) call “exit politics”, are essential in the emergence of user’s organised actions. In their quest for expression and engagement, users reflexively produce a sense of being “outside”: Outside of the legibility of the legal system and its figures and fictions, outside of the approachability of the mental health system, outside of the temporality of its National Plan and outside of its descriptive ambitions. Even direct opposition is dismissed as mere reaction, favoring what could be called a politics of incommensurability: not just against or in contradiction to any given order, but not mapping into it or alongside it (Lambek 2012).

“Being outside” has specific connotations in the case of mental health service users, considering that many of them had struggled to get out of mental health institutions in the past. Even in community mental health settings, users become subjects of intensive, prolonged, incongruent and often damaging practices of description stemming from diagnostic models and procedures (Ben-Zeev, Young, and Corrigan 2010; Moncrieff 2010; Rose 2006). Using Rancière’s terminology, this practice of description is the fundamental action of psychiatric “police” after the asylum. Incommensurability as “politics” represents the simultaneous suspension of that process of identification, its replacement with a new regime of collective self-identification and the rejection of any auxiliary position in relation to the mental health system. The findings reveal how ALM went through this *political* process. For Rancière:

“Politics begins when those who were destined to remain in the domestic and invisible territory of work and reproduction, and prevented from doing ‘anything else’, take the time that they ‘have not’ in order to affirm that they belong to a common world. It begins when they make the invisible visible, and make what was deemed to be the mere noise of suffering bodies heard as a discourse concerning the ‘common’ of the community. Politics creates a new form, as it were, of dissensual ‘commonsense” (2010:147)

Mental health systems see as their goal the alleviation of mental illness and suffering. This reduces the complexity of their potentially immense number of interlocutors and also defines the scope and characteristics of that interlocution (Montenegro and Cornish 2017). A user is a user because it needs something from the system, and the system approaches users on this basis. The notion of a politics of incommensurability points to the conversations, decisions and gestures by which users and ex-users collectively dispute their intelligibility and approachability. In the terminology of Rancière, it is the process by which a group break with its “destiny” -understood as their expected role- in order to define *their own* role.

#### Rethinking Participation in Mental Health Systems

As indicated in the introduction, the call for users’ involvement continues to influence policy making, especially in the English speaking world but increasingly in countries in the “global south” (Lempp et al. 2017; Semrau et al. 2016). Reproducing a technical, top-down view of “involvement”, these evaluations see the role of users as one of integration and continuity with the roles of mental health systems, with no tension or opposition between users and mental health systems, and with no apparent differentiation between family/caregiver and user participation. Under the umbrella goal of “scaling up” services (Semrau et al. 2015) and consonant with the ambitions of contemporary global mental health (Eaton et al. 2011; Patel et al. 2014) these and other studies are setting the standard of evaluation of service-user participation and advocacy in other parts of the world (World Health Organization 2013).

This case study confronts a reductionist and decontextualised approach to involvement by identifying practices of reflexivity and incommensurability as critical elements in the emergence of mental health users’ and ex-users’ activism. Incommensurability directly undermines the “measurability” of users’ influence and presence, especially when observed as a component of a modern mental health system.

An undifferentiated call for participation needs to be supplemented by approaches that embrace the variety of forms taken by mental health users’ activism in different settings and regions. This means placing the emergence of self-initiated collective action in its own, specific socio-political milieu and historical background. It also means that the disputes, disagreements and opposition should be viewed as central aspects in the analysis of politics and participation in this field (Carr 2007) and not simply as “risks” to be avoided. Finally, closer attention to how users and activist make sense of their own role opens analytical ways to critically understand the shifting expectations, dispositions and ambitions of mental health policy, at national and global levels.

## Footnotes

1 In Chile, “chaquetero” is someone that expresses harsh criticism out of envy for someone else’s achievements or position.

2 By law each health service in the public health system has a “Local Development Council” acting in representation of the communities served.

3 It is important to note that semantically, the word loco is closer to “crazy” than to “mad”. Specifically, the word “loco” does not denotes anger.

## References

[CR1] Abram S., Weszkalnys G. (2011). Introduction: Anthropologies of planning—Temporality, imagination, and ethnography. Focaal.

[CR2] Archer M.S. (2007). Making our Way Through the World: Human Reflexivity and Social Mobility.

[CR3] Archer M.S., Powell C.J., Dépelteau F. (2013). Collective Reflexivity: A Relational Case for It. Conceptualizing Relational Sociology: Ontological and Theoretical Issues.

[CR4] Bacqué M.-H., Rey H., Sintomer Y. (2004). Gestion de Proximité et Démocratie Participative: Une Perspective Comparative.

[CR6] Barnes, M. 1999a Unequal Partners: User Groups and Community Care. Bristol: Policy Press.

[CR7] Barnes, M. 1999b Users as Citizens: Collective Action and the Local Governance of Welfare. Social Policy and Administration, 33(1):73–90.

[CR8] Ben-Zeev D., Young M.A., Corrigan P.W. (2010). DSM-V and the Stigma of Mental Illness. Journal of Mental Health.

[CR9] Beresford P. (2010). Public Partnerships, Governance and User Involvement: A Service User Perspective. International Journal of Consumer Studies.

[CR10] Brody E.B. (2004). The World Federation for Mental Health: Its Origins and Contemporary Relevance to WHO and WPA Policies. World Psychiatry.

[CR11] Brosnan L. (2012). Power and Participation: An Examination of the Dynamics of Mental Health Service-User Involvement in Ireland. Studies in Social Justice.

[CR12] Cabalin C. (2012). Neoliberal Education and Student Movements in Chile: Inequalities and Malaise. Policy Futures in Education.

[CR13] Caldas de Almeida J.M. (2005). Estrategias de Cooperación Técnica de la Organización Panamericana de la Salud en la Nueva Fase de la Reforma de los Servicios de Salud Mental en América Latina y el Caribe. Revista Panamericana de Salud Pública.

[CR15] Campbell P., Heller T., Reynolds J., Gomm R., Muston R., Pattison S. (1996). The History of the User Movement in the United Kingdom. Mental Health Matters: A Reader.

[CR16] Carr S. (2007). Participation, Power, Conflict and Change: Theorizing Dynamics of Service User Participation in the Social Care System of England and Wales. Critical Social Policy.

[CR17] Ceriani, L., J. Obiols, and A. Stolkiner 2010 Potencialidades y obstáculos en la construcción de un nuevo actor social: Las organizaciones de usuarios. In II Congreso Internacional de Investigación y Práctica Profesional en Psicología XVII Jornadas de Investigación Sexto Encuentro de Investigadores en Psicología del MERCOSUR. Facultad de Psicología-Universidad de Buenos Aires.

[CR18] Connor, B. 2014 The Centrality of Disagreement. Doctoral Dissertations May 2014-Current. Retrieved from http://scholarworks.umass.edu/dissertations_2/170

[CR19] Cooper J. (2018). Unruly Affects: Attempts at Control and All That Escapes from an American Mental Health Court. Cultural Anthropology.

[CR20] Contandriopoulos D. (2004). A Sociological Perspective on Public Participation in Health Care. Social Science and Medicine.

[CR21] Crossley N. (2005). How Social Movements Move: From First to Second Wave Developments in the UK Field of Psychiatric Contention. Social Movement Studies.

[CR22] Crossley, N. (2006a). Contesting psychiatry social movements in mental health. London: Routledge.

[CR23] Crossley, N. (2006b). The field of psychiatric contention in the UK, 1960–2000. Social Science & Medicine, 62(3), 552–563. 10.1016/j.socscimed.2005.06.016 10.1016/j.socscimed.2005.06.016 16039030

[CR25] del Campo A. (2016). Theatricalities of Dissent Human Rights, Memory, and the Student Movement in Chile. Radical History Review.

[CR31] de Freitas, C.S.S. 2011 Participation in Mental Health Care by Ethnic Minority Users: Case Studies from the Netherlands and Brazil. (Self-Published PhD Thesis). University of Utrecht, S.l.

[CR26] Dewey J. (1926). The Historic Background of Corporate Legal Personality. The Yale Law Journal.

[CR27] Dornhof S. (2011). Regimes of Visibility: Representing Violence Against Women in the French Banlieue. Feminist Review.

[CR28] Eaton J., McCay L., Semrau M., Chatterjee S., Baingana F., Araya R., Ntulo C., Thornicroft G., Saxena S. (2011). Scale Up of Services for Mental Health in Low-Income and Middle-Income Countries. The Lancet.

[CR29] El Enany N., Currie G., Lockett A. (2013). A Paradox in Healthcare Service Development: Professionalization of Service Users. Social Science and Medicine.

[CR30] Everett B. (2000). A Fragile Revolution Consumers and Psychiatric Survivors Confront the Power of the Mental Health System.

[CR32] Harrison S., Mort M. (1998). Which Champions, Which People? Public and User Involvement in Health Care as a Technology of Legitimation. Social Policy and Administration.

[CR33] Jazeel T. (2015). Political Aesthetics and Embodiment: Sung Protest in Post-apartheid South Africa. Journal of Material Culture.

[CR34] Klee S. (2013). Rancière Against the Cuts. Third Text.

[CR35] Knoblauch H. (2005). Focused Ethnography. Forum Qualitative Sozialforschung/Forum.

[CR36] Lambek M., Fassin D. (2012). Religion and Morality. A Companion to Moral Anthropology.

[CR37] Lempp H., Abayneh S., Gurung D., Kola L., Abdulmalik J., Evans-Lacko S., Semrau M., Alem A., Thornicroft G., Hanlon C. (2017). Service User and Caregiver Involvement in Mental Health System Strengthening in Low- and Middle-Income Countries: a Cross-Country Qualitative Study. Epidemiology and Psychiatric Sciences.

[CR38] Lewis L. (2014). User Involvement in Mental Health Services: A Case of Power over Discourse. Sociological Research Online.

[CR39] Locos por Nuestros Derechos (2015). Manual de Derechos en Salud Mental.

[CR40] Luhmann N. (1995). Social Systems.

[CR41] Massey D., Massey D., Allen J., Sarre P. (1999). Spaces of Politics. Human Geography Today.

[CR42] May T. (2008). Equality Among the Refugees: A Rancièrean View of Montréal’s Sans-Status Algerians. Anarchist Studies.

[CR43] Minkowitz, T. 2013 CRPD Advocacy by the World Network of Users and Survivors of Psychiatry: The Emergence of an User/Survivor Perspective in Human Rights. SSRN Working Paper Series. 10.2139/ssrn.2326668.

[CR44] Minoletti A., Sepúlveda R., Horvitz-Lennon M. (2012). Twenty Years of Mental Health Policies in Chile: Lessons and Challenges. International Journal of Mental Health.

[CR45] Minoletti A., Toro O., Alvarado R., Carniglia C., Guajardo A., Rayo X. (2015). A Survey About Quality of Care and Users Rights in Chilean Psychiatric Services. Revista Médica de Chile.

[CR46] Moncrieff J. (2010). Psychiatric Diagnosis as a Political Device. Social Theory and Health.

[CR47] Montalbán A. (2013). Retomando el Debate Sobre la Organización de la Atención Psiquiátrica y Salud Mental en Uruguay. Revista de Psiquiatría del Uruguay.

[CR48] Montenegro C.R., Cornish F. (2015). ‘It is not the State’s fault that we have a person like this’: Relations, Institutions and the Meaning of ‘rights’ to Carers of People with Psychosocial Disabilities in Chile. Global Mental Health.

[CR49] Montenegro C.R., Cornish F. (2017). Historicising Involvement: the Visibility of User Groups in the Modernisation of the Chilean Mental Health System. Critical Public Health.

[CR50] Morrison L.J. (2005). Talking Back to Psychiatry: the Psychiatric Consumer/Survivor/Ex-Patient Movement.

[CR51] MUMS Chile n.d. MUMS Chile-25 Años de Historia. Retrieved 6 January 2017 from http://mums.cl/historia/

[CR52] Newman J. (2009). Publics, Politics, and Power: Remaking the Public in Public Services.

[CR53] Noorani T. (2013). Service User Involvement, Authority and the ‘expert-by-experience’ in Mental Health. Journal of Political Power.

[CR54] Papadopoulos D., Stephenson N., Tsianos V. (2008). Escape Routes: Control and Subversion in the Twenty-First Century.

[CR55] Patel V., Minas I.H., Cohen A., Prince M. (2014). Global Mental Health: Principles and Practice.

[CR56] Perez O., Teubner G. (2006). Paradoxes and Inconsistencies in the Law.

[CR57] Pérez Soto C. (2012). Una Nueva Antipsiquiatría: Crítica y Conocimiento de las Técnicas de Control Psquiátrico.

[CR58] Pilgrim D., Bell A., Lindley P. (2005). Protest and Co-option: The Voice of Mental Health Service Users. Beyond the Water Towers: the Unfinished Revolution in Mental Health Services.

[CR59] Rancière J. (1999). Disagreement: Politics and Philosophy.

[CR60] Rancière, J. 2010 Dissensus: on Politics and Aesthetics. (S. Corcoran, Trans.). New York: Continuum.

[CR61] Renedo A., Marston C. (2015). Spaces for Citizen Involvement in Healthcare: An Ethnographic Study. Sociology.

[CR62] Rose N. (2006). Disorders Without Borders? The Expanding Scope of Psychiatric Practice. BioSocieties.

[CR63] Scott J.C. (1990). Domination and the Arts of Resistance: Hidden Transcripts.

[CR64] Semrau M., Evans-Lacko S., Alem A., Ayuso-Mateos J.L., Chisholm D., Gureje O., Thornicroft G. (2015). Strengthening Mental Health Systems in Low- and Middle-Income Countries: The Emerald programme. BMC Medicine.

[CR65] Semrau M., Lempp H., Keynejad R., Evans-Lacko S., Mugisha J., Raja S., Lamichhane J., Alem A., Thornicroft G., Hanlon C. (2016). Service User and Caregiver Involvement in Mental Health System Strengthening in Low- and Middle-Income Countries: Systematic Review. BMC Health Services Research.

[CR66] Susser E., Valencia E., Conover S., Felix A., Tsai W.Y., Wyatt R.J. (1997). Preventing Recurrent Homelessness Among Mentally Ill Men: a ‘critical time’ Intervention After Discharge from a Shelter. American Journal of Public Health.

[CR67] Teubner G., Perez O., Teubner G. (2006). Dealing with Paradoxes of Law: Derrida, Luhmann, Wiethölter. Paradoxes and Inconsistencies in the Law.

[CR68] Wall S.S. (2014). Focused Ethnography: A Methodological Adaptation for Social Research in Emerging Contexts. Forum Qualitative Sozialforschung/Forum: Qualitative Social Research.

[CR69] Wallcraft J.A.N., Amering M., Freidin J., Davar B., Froggatt D., Jafri H., Javed A., Katontoka S., Raja S., Rataemane S., Steffen S. (2011). Partnerships for Better Mental Health Worldwide: WPA Recommendations on Best Practices in Working with Service Users and Family Carers. World Psychiatry.

[CR70] World Health Organization (2013). WHO Mental Health Action Plan 2013–2020.

[CR71] Zaldúa, G., M. Bottinelli, M.B. Sopransi, R. Longo, M. Nabergoi, A. Tisera, M.M. Lenta, R. Moschella, M. Freire 2012 A Un Año de la Ley Nacional de Salud Mental: Nuevos Desafíos Para Las Políticas Públicas, Los Dispositivos y Las Prácticas. Presented at the Congreso Regional de la Sociedad Interamericana de Psicología.

